# Additively manufactured biodegradable porous magnesium implants for elimination of implant-related infections: An *in vitro* and *in vivo* study

**DOI:** 10.1016/j.bioactmat.2021.06.032

**Published:** 2021-07-06

**Authors:** Kai Xie, Nanqing Wang, Yu Guo, Shuang Zhao, Jia Tan, Lei Wang, Guoyuan Li, Junxiang Wu, Yangzi Yang, Wenyu Xu, Juan Chen, Wenbo Jiang, Penghuai Fu, Yongqiang Hao

**Affiliations:** a Shanghai Key Laboratory of Orthopaedic Implants, Department of Orthopaedic Surgery, Shanghai Ninth People's Hospital, Shanghai Jiao Tong University School of Medicine, Shanghai, 200011, China; b National Engineering Research Center of Light Alloy Net Forming & State Key Laboratory of Metal Matrix Composite, Shanghai Jiao Tong University, Shanghai, 200240, China; c Musculoskeletal Tumor Center, Peking University People's Hospital, 100044, Beijing, China; d Department of Orthopedics, The First Affiliated Hospital of USTC, Division of Life Sciences and Medicine, University of Science and Technology of China, Hefei, Anhui, 230001, China; e Clinical and Translational Research Center for 3D Printing Technology, Shanghai Ninth People's Hospital, Shanghai Jiao Tong University School of Medicine, Shanghai, 200011, China

**Keywords:** Magnesium implants, 3D printing, Implant-related infections, Antibacterial activity

## Abstract

Magnesium (Mg) alloys that have both antibacterial and osteogenic properties are suitable candidates for orthopedic implants. However, the fabrication of ideal Mg implants suitable for bone repair remains challenging because it requires implants with interconnected pore structures and personalized geometric shapes. In this study, we fabricated a porous 3D-printed Mg-Nd-Zn-Zr (denoted as JDBM) implant with suitable mechanical properties using selective laser melting technology. The 3D-printed JDBM implant exhibited cytocompatibility in MC3T3-E1 and RAW267.4 cells and excellent osteoinductivity *in vitro*. Furthermore, the implant demonstrated excellent antibacterial ratios of 90.0% and 92.1% for methicillin-resistant *S. aureus* (MRSA) and *Escherichia coli*, respectively. The 3D-printed JDBM implant prevented MRSA-induced implant-related infection in a rabbit model and showed good *in vivo* biocompatibility based on the results of histological evaluation, blood tests, and Mg^2+^ deposition detection. In addition, enhanced inflammatory response and TNF-α secretion were observed at the bone-implant interface of the 3D-printed JDBM implants during the early implantation stage. The high Mg^2+^ environment produced by the degradation of 3D-printed JDBM implants could promote M1 phenotype of macrophages (Tnf, iNOS, Ccl3, Ccl4, Ccl5, Cxcl10, and Cxcl2), and enhance the phagocytic ability of macrophages. The enhanced immunoregulatory effect generated by relatively fast Mg^2+^ release and implant degradation during the early implantation stage is a potential antibacterial mechanism of Mg-based implant. Our findings indicate that 3D-printed porous JDBM implants, having both antibacterial property and osteoinductivity, hold potential for future orthopedic applications.

## Highlights

• Porous JDBM implants promising mechanical properties was fabricated by selective laser melting.
• 3D-printed JDBM implant exhibited excellent antibacterial property, osteoinductivity, and biocompatibility.
• Temporally enhanced immunoregulatory effect in early stage was a potential antibacterial mechanism of Mg-based implant.

## Graphical abstract

**Figure:**
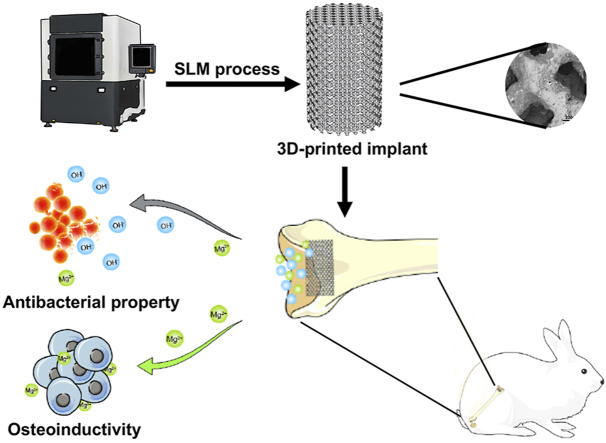

## 1 Introduction

Metal implants are widely used in orthopedic surgery, and implant-related infections are one of the most challenging complications in orthopedic surgery, and these infections are not rare. In the United States, for example, the infection rate of orthopedic implants is approximately 4.3% [1], while the incidence of infections exceeds 20% for complex open fractures [2]. According to previous reports, Gram-positive cocci, such as *Staphylococcus aureus* (*S. aureus*) and coagulase-negative staphylococci, are the most common causative organisms. The traditional treatment options for implant-related infections include surgical debridement, long-term systemic antibiotic therapy, and local antibiotic therapy [3]. Even though the success rates of these treatment options range from 70 to 90% [4], these procedures inevitably lead to extensive surgery, heavy financial burden, and a high incidence of devastating complications. Therefore, an implant with antibacterial properties is urgently required to prevent and treat such infections [[5], [6], [7]].

Considering their biocompatibility, osteoinductivity, and mechanical properties with respect to natural bone, biodegradable magnesium (Mg) and Mg alloys are potential candidates for orthopedic implants. Over the past few years, these biodegradable Mg and Mg alloys have been reported to have antibiofilm properties [8,9]. The alkaline environment produced during the degradation of Mg alloys has a broad-spectrum bactericidal ability [10,11]. Qin et al. [12] reported that Mg-Nd-Zn-Zr alloys with enhanced corrosion resistance and biocompatibility effectively inhibited both Gram-positive (*S. aureus* and *Staphylococcus epidermidis*) and Gram-negative (*Escherichia coli, [E. coli]*) bacteria, and prevented implant-related infection in a rat model. In addition to direct killing effects, the rapid increase in pH and Mg^2+^ concentration in the microenvironment during degradation prevented biofilm formation by downregulating the expression of biofilm-related genes of methicillin-resistant *S. aureus* (MRSA) [13,14]*.* Therefore, as biodegradable materials with both antibacterial and osteogenic properties, Mg alloys have excellent application potential to be used as orthopedic implants.

However, fabrication of biodegradable Mg alloy implants depends primarily on casting technologies and computer numerical control machines, which are limited by long manufacturing cycles, shape limitations, and raw material waste. Porous metallic implants with suitable porosity values are possible bone substitutes due to their adjustable mechanical strength and ability to allow bone and blood vessel ingrowth [15]. The traditional approach to the manufacturing of porous Mg implants was based on a soluble space holder (which acted as a temporary template) infiltrated with Mg and then dissolved to create a pore structure following solidification [16,17]. However, the soluble template approach always resulted in random porosity, pore size, and mechanical strength and did not allow control over the precise shape of the implant [18]. Metal additive manufacturing techniques, such as selective laser melting (SLM) and electron beam melting, offer novel approaches to achieve rapid manufacturing in developing clinical implants with complex shapes and internal structures at high resolution. However, the application of additive manufacturing to biodegradable Mg alloy implants remains challenging and relatively dangerous due to the highly active chemical properties of Mg [19]. To the best of our knowledge, the *in vivo* biological behavior of such implants has not been reported.

In our previous animal study, enhanced immunological response and inflammatory cell infiltration were observed in the peri-implant bone tissue during the first week after the implantation of 3D-printed porous Mg implants, decreasing gradually over time. This could have been caused by the rapid Mg^2+^ release from the 3D-printed porous Mg implants during their degradation. Coincidentally, a previous study reported that the rapid degradation of Mg alloys was associated with macrophage infiltration and enhanced inflammatory response in the periprosthetic tissue [20]. It is known that implant-related infections are complex processes that involve interactions between the implant, bacteria, and host immune system [21], and macrophages play a prominent role in the host's immune response against *S. aureus* infection [22]. In response to bacterial infection, macrophages switch to classically activated M1 phenotype and then phagocytose and destroy the invading bacteria. However, *S. aureus* has developed numerous methods to manipulate the switching of the macrophages from the microbicidal M1 phenotype to the anti-inflammatory M2 phenotype, preventing the phagocytosis-mediated killing of bacteria [23]. Liu et al. [24] reported that increased M1 polarization and inflammatory cytokine release stimulated by the surface-engineered polyetheretherketone implant showed immunoregulatory antibacterial activity against MRSA*.* Therefore, we hypothesized that temporally enhanced immunological response in the peri-implant tissue around the 3D-printed Mg implant could be beneficial for the inhibition and treatment of implant-related infection.

Given these observations, we fabricated porous Mg alloy implants using the SLM technology and evaluated the antibacterial properties and biocompatibility of the 3D-printed Mg alloy implant *in vitro* and *in vivo*. The effect of increased Mg^2+^ concentration following implant degradation on macrophages was determined by evaluating the macrophage phenotype, phagocytic ability, and histological evaluation. The *in vitro* cytocompatibility and *in vivo* biosafety of the implant were also determined.

## 2 Materials and methods

### 2.1 Materials and extract preparation

#### 2.1.1 Scaffold manufacturing

**Fig. 1:**
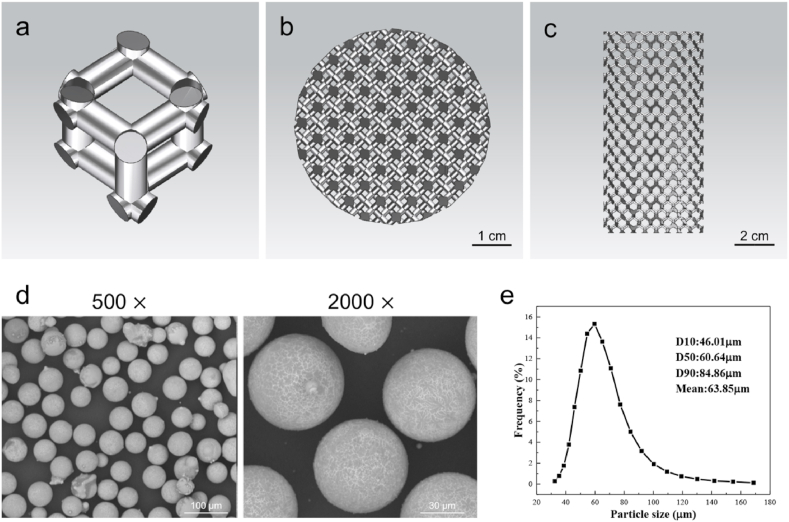
Design of the 3D-printed JDBM implant and characterization of Mg–Nd–Zn–Zr powder. a) Diamond unit cell. b) Top view of the computer-aided design model. c) Lateral view of the computer-aided design model. d) SEM image of the Mg–Nd–Zn–Zr powder. e) Particle size distribution of the Mg–Nd–Zn–Zr powder.

The scaffold with diamond unit cells (Fig. 1a–c) was designed using Materialise Magics (version 15.0, Materialise, Leuven, Belgium). The designed pore size and porosity of 3D-printed Mg–Nd–Zn–Zr (denoted as JDBM) implant were 300–400 μm and 80.0%, respectively. The scaffold was facilitated with SLM using a 3D-printing machine (SLM150, ZRapid Tech Co., LTD, China). Magnesium alloy (Mg-3.16Nd-0.18Zn-0.41Zr) powder was prepared using centrifugal atomization (Fig. 1d). The distribution of the powder particle size is presented in Fig. 1e. The mean particle size of the magnesium alloy powder was 63.9 ± 14.5 μm, as determined using a laser particle size analyzer (S3500, Microtrac Inc., USA). The specific experimental procedure was as follows. The powder bed and Mg–RE substrate were pre-heated at 100 °C for 1 h before printing. During the SLM process, the working chamber was filled with argon, and the oxygen was controlled below 500 ppm. The processing parameters used were as follows: 80 W laser power, 450 mm/s scanning speed, 20 μm layer thickness, and 70 μm hatch spacing. The scanning direction was rotated by 73° between the neighboring layers. The chemical composition of 3D-printed JDBM alloy was identified as Mg-3.24Nd-0.21Zn-0.44Zr, where the content of Nd, Zn, and Zr in the alloy was higher compared to the powder, possibly due to the evaporation of Mg during the SLM process. A perchloric acid alcohol solution, consisting of 10 vol% perchloric acid and 90 vol% C_2_H_5_OH, was used as a polishing solution. The post-processing treatment methods for 3D-printed JDBM implants are presented in Supplementary Material 1.1.

#### 2.1.2 Material and mechanical characterization

The surface morphology of the 3D-printed JDBM implants was evaluated using scanning electron microscopy (SEM). SEM images were analyzed using the Image J 1.48 software (National Institutes of Health, USA) to determine the implant pore size. The solid-solution-treated JDBM implants were scanned by Micro Computed Tomography (μCT, SCANCO Medical AG, Bassersdorf, Switzerland) to calculate the porosity of the implant. The microstructure of the implant was determined using optical microscopy (OM, Zeiss Axio ObserverA1) and SEM (NOVA NanoSEM 230). After solid solution treatment, the JDBM implant was subjected to a room temperature compression test at a crosshead speed of 1 mm/min. The yield strength, compressive strength, and elastic modulus were obtained.

#### 2.1.3 In vitro degradation and Mg ion release measurements

The *in vitro* degradation properties of 3D-printed JDBM implants were determined as previously described with minor modifications [25,26]. Briefly, the 3D-printed JDBM implants were incubated in simulated body fluid at room temperature for 7 d. The Mg^2+^ concentration and the hydrogen release were determined at 24-h intervals. The degradation rate (r) of 3D-printed JDBM implants was calculated by the following equation [27]:

$$r=\frac{Material\;mass\;before\;degradation-Material\;mass\;after\;degradation}{Immersion\;time}$$

#### 2.1.4 Preparation of sample extracts

All sample extracts were prepared in accordance with ISO 10993-5 and ISO 10993-12. For *in vitro* experiments, sample extracts were prepared by incubating samples in Alpha-Modified Eagle Medium (Hyclone) or Dulbecco's Modified Eagle Medium (Hyclone) supplemented with 10% fetal bovine serum (Gibco) and 1% streptomycin-penicillin (Hyclone) at 37 °C in a humidified atmosphere with 5% CO_2_ for 3 d. The sample extract was diluted to specific concentrations (50, 25, and 12.5%) with the corresponding culture medium for further use. For antibacterial experiments, JDBM samples were immersed in tryptic soy broth (TSB) at 37 °C in a humidified atmosphere with 5% CO_2_ for 3 d. The pH of sample extract was adjusted to 7.4 using either HCl or NaOH.

### 2.2 In vitro cytocompatibility and osteogenic differentiation

#### 2.2.1 Cell viability

MC3T3-E1 and RAW 264.7 cells were purchased from The Cell Bank of the Type Culture Collection of Chinese Academy of Sciences (Shanghai, China). Sample extracts that were diluted two, four, and eight times were prepared for further cytotoxicity tests, as previously described [28,29]. The detailed methods for cytocompatibility experiments are described in Supplementary Material 1.2.

#### 2.2.2 Osteogenic differentiation

The osteogenic differentiation of the MC3T3-E1 cells was evaluated by alkaline phosphatase (ALP) and alizarin red staining. The MC3T3-E1 cells were seeded in 24-well plates at a density of 5 × 10^4^/well. After cells reached ~70% confluence, the culture medium was replaced with media containing different concentrations of extracts (100, 50, 25, and 12.5%) and supplemented with 10 mM β-glycerophosphate, 50 mM ascorbic acid, and 100 nM dexamethasone (Sigma, USA) to promote osteogenic differentiation. ALP staining was performed, as previously described, on day 7 [30]. To evaluate the formation of calcified nodules, cultures were stained with alizarin red on day 21.

### 2.3 Evaluation of antibacterial properties *in vitro*

#### 2.3.1 Bacterial culture preparation

MRSA (ATCC43300) and *E. coli* (ATCC25922) were incubated in TSB overnight at 37 °C. Next, bacteria were resuspended in TSB and sample extracts. Bacterial suspensions were diluted to 1 × 10^6^ CFU/mL for further use.

#### 2.3.2 Determination of antibacterial activity

A suspension (100 μL/well) containing 1 × 10^6^ CFU/mL bacteria was added to each well of a 96-well plate. The plates were then incubated at 37 °C for 1, 3, 6, 12, and 24 h. After incubation, the bacterial growth in TSB extracts (n = 5) was determined by measuring absorbance at 600 nm using a microplate reader (Infinite 200, Tecan Trading AG, Switzerland) [31]. The antibacterial activity of sample extracts was determined by antibacterial ratio based on the absorption of optical density (OD), and was quantified using the following equation:

$$Antibacterial\;ratio\;\left(\%\right)=\frac{OD\;value\;of\;control\;group-OD\;value\;of\;experimental\;group}{OD\;value\;of\;control\;group}$$

For spread plate analysis, the bacterial suspensions were serially diluted, spread on tryptone soy agar (TSA), and then incubated at 37 °C. The bacterial colonies were counted after 24 h of incubation (n = 5).

#### 2.3.3 Determination of bacterial biofilm formation

The bacterial biofilm formation in different sample extracts was determined by LIVE/DEAD staining and crystal violet staining as previously described [32]. The detailed methods are presented in Supplementary Material 1.3.

### 2.4 In vivo anti-infective experiments

The *in vivo* antibacterial properties of 3D-printed JDBM implants were evaluated using a rabbit model by performing radiographic analysis and histological evaluation (see detailed description in Supplementary Material 1.4).

### 2.5 Effects of high Mg^2+^ environment on macrophage function

#### 2.5.1 Transcriptomic analysis

The effect and mechanism of Mg^2+^ on macrophage polarization were investigated by transcriptomic analysis. The RAW 264.7 cells (2 × 10^5^ per well) were seeded in 6-well plates and incubated at 37 °C in a humidified atmosphere with 5% CO_2_. After 24-h incubation, the culture medium was replaced with sample extracts (pH = 7.4). After three days of incubation, cells were collected, and total RNA was isolated. Transcriptomic analysis was performed by Applied Protein Technology Co. Ltd. Differential expression analysis was performed using the DESeq2 R package (1.16.1). Gene ontology (GO) and Kyoto Encyclopedia of Genes and Genomes (KEGG) enrichment analyses were performed to determine the potential biological functions of differentially expressed genes.

#### 2.5.2 Real-time reverse transcription-polymerase chain reaction (RT-PCR)

Cells were cultured with sample extracts (pH = 7.4) for 72 h and the total RNA from RAW 264.7 cells was extracted using an RNeasy mini kit (Cyagen Biosciences). The total RNA was reverse transcribed to complementary DNA with a PrimeScript RT reagent kit (TaKaRa, Japan) according to the manufacturer's instructions, and the RT-PCR was performed on a QuantStudio 6 flex real-time PCR system (Applied Biosystems, France) using a TB Green Premix Ex Taq (TaKaRa, Japan). Commercially synthesized primers (Sangon Biotech Co., Ltd., China) are listed in Table S1 (Supporting Information). Cells cultured in DMEM culture medium supplemented with 10% fetal bovine serum (Gibco) and 1% streptomycin-penicillin (Hyclone) were considered the control group.

#### 2.5.3 Flow cytometry

The effects of Mg^2+^ on macrophage polarization were further evaluated by flow cytometry. The RAW 264.7 cells were seeded in 6-well plates at a density of 2 × 10^5^/well and cultured in DMEM culture medium for 24 h. Next, the culture medium was replaced with DMEM containing sample extracts (pH = 7.4). After 72 h of stimulation, cells from different groups were collected for further experiments. CD86 and CD206 were used as the markers of M1 and M2 phenotypes, respectively. PE-Cy™7 Rat IgG2a, κ Isotype Control (BD, CA, USA), and rat IgG2b κ Isotype control (eBioscience, CA, USA) were used to distinguish the positive cells. Flow cytometry was performed using a FACScan flow cytometer (BD, CA, USA).

#### 2.5.4 Enzyme-linked immunosorbent assay (ELISA)

The RAW 264.7 cells were cultured with sample extracts (pH = 7.4) for 72 h. The culture medium was collected and centrifuged. The concentrations of tumor necrosis factor alpha (TNF-α) and inducible nitric oxide synthase (iNOS) were detected using ELISA kits according to the manufacturer's instructions.

#### 2.5.5 Phagocytosis detection assay

The MRSA was labeled using 5(6)-carboxy-fluorescein diacetate succinimidyl ester (CFDA-SE, Sigma, USA) as previously described [24]. RAW 264.7 cells were seeded in 6-well plates at a density of 2 × 10^5^/well and cultured in supplemented DMEM culture medium for 24 h. Next, the culture medium was replaced with the DMEM containing sample extracts (pH = 7.4). After 72 h of stimulation, cells from different groups were collected for further experiments. RAW 264.7 cells (1 × 10^6^ cells) were mixed with MRSA (1 × 10^7^ CFU, MOI: 10). The suspension was incubated at 37 °C for 1 h. After 1 h, the cells were centrifuged at 200×*g* for 1 min to remove the non-phagocytosed bacteria [33]. For spread plate analysis, cells were treated with 0.2% Triton X-100 to release phagocytosed MRSA [24]. The bacterial suspension was serially diluted and seeded on TSA. After incubating for 24 h, the bacterial colonies were counted. For fluorescence microscope observations, cells were further plated in 24-well plates (2 × 10^5^/well), incubated at 37 °C for 15 min, and then fixed with 4% paraformaldehyde at room temperature for 10 min. To detect F-actin, fixed RAW 264.7 cells were stained with rhodamine phalloidin (Cytoskeleton, Inc.) and observed using a fluorescent microscope (Leica Microsystems, Heidelberg, Germany).

### 2.6 Biosafety evaluation

Blood samples were collected prior to euthanasia four weeks after implantation. The serum magnesium concentrations, routine blood parameters, liver function test, and renal function test were performed according to the manufacturer's instructions (n = 5). After euthanasia, the liver, kidney, heart, spleen, and lung were immediately obtained from each group, and Mg^2+^ deposits in those organs were determined using inductively coupled plasma mass spectrometry [14]. In addition, the histological evaluation of the liver, kidney, heart, spleen, and lung morphology was performed after the hematoxylin and eosin (HE) staining.

### 2.7 Statistical analysis

The sample size was three in current study, except when indicated otherwise. Student's *t*-test and one-way analysis of variance were used to determine the variance using the SPSS version 23.0 software (IBM Corp.). A two-sided p-value <0.05 was considered statistically significant.

## 3 Results

### 3.1 Characterization of the implant

**Fig. 2:**
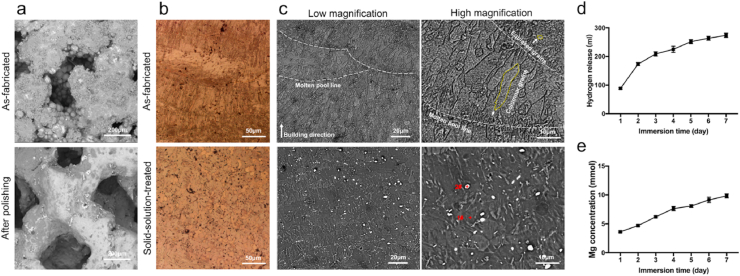
Characterization and degradation behavior of the 3D-printed JDBM implant. a) Surface morphology of the 3D-printed JDBM implants. b) Optical microscope images of 3D-printed JDBM implants. c) SEM images of as-fabricated and solid-solution-treated 3D-printed JDBM implant observed from side surfaces. d) Hydrogen release after immersing for 1–7 days. e) Mg^2+^ concentrations after immersing for 1–7 days.

Fig. 2a shows that numerous powder particles were observed on the strut surface in the as-fabricated implant, and the electrochemical polishing process was an effective method to partially remove the JDBM powders from the surface of 3D-printed JDBM implants. The porosity of as-fabricated implant before polishing was 32.1 ± 1.3%. The mean pore size and mean porosity of the solid-solution-treated implant were 324.6 ± 25.7 μm and 52.1 ± 1.6%, respectively. The OM and SEM images of the 3D-printed JDBM implant observed from side surface are presented in Fig. 2b and c. In the as-fabricated implant, fish-scale-shaped melt pools were clearly revealed and were composed of columnar and equiaxed grains. The columnar grains grew along the inside of the molten pool, which was more consistent with the direction of heat dissipation. The equiaxed grains were formed on the boundary of the molten pool (Fig. 2c), where the cooling rate was considered to be significantly higher compared to the inside of the molten pool. Fine white dot particles were also observed on the grain interiors, which were probably the Mg_12_Nd phase formed during the solidification process, as suggested by the X-ray diffraction (XRD) result (Fig. S1). After heat treatment, the boundaries of the molten pools and columnar grains were invisible, and only larger equiaxed grains and white dot particles were observed. The average grain size of the equiaxed grains increased to 20–25 μm, whereas the volume of the white dots greatly exceeded that in the as-fabricated condition. The composition of white dots (indicated by “plus” signs in Fig. 2c) was further analyzed by energy-dispersive X-ray spectroscopy (EDS) and the results are shown in Table 1. Dot #1 was considered as the Mg matrix due to the low Nd content. The Nd content of dot #2 was much higher compared to the Mg_12_Nd eutectic phase (33.1 wt%) and was considered to be a rare-earth hydride (NdH_2_), as identified by the XRD (Fig. S2). The compressive yield strength, ultimate compressive strength, and elastic modulus of the 3D-printed JDBM implant after solution treatment were 54.80 ± 6.43 MPa, 97.13 ± 7.58 MPa, and 1.98 ± 0.02 GPa, respectively.

**Table 1:** Results of Energy-dispersive X-ray spectroscopy analysis.

	Mg	Nd	O
1#	95.77%	1.09%	3.14%
2#	15.53%	79.72%	4.75%

### 3.2 In vitro degradation

The 3D-printed JDBM implant was immersed in SBF for 7 d. The degradation rate of 3D-printed JDBM implant was presented in Fig. S3. The short-term degradation rate was estimated as 0.039 ± 0.003 g/day in SBF after 7 d of immersion. Our results demonstrated that the degradation of 3D-printed JDBM implant (hydrogen release) decreased with the immersion time (Fig. 2d). As determined by the ICP-AES analysis, the Mg^2+^ concentration increased rapidly on day 1 (Fig. 2e), creating a potentially beneficial antibacterial effect of the 3D-printed JDBM implant.

### 3.3 In vitro cytocompatibility

**Fig. 3:**
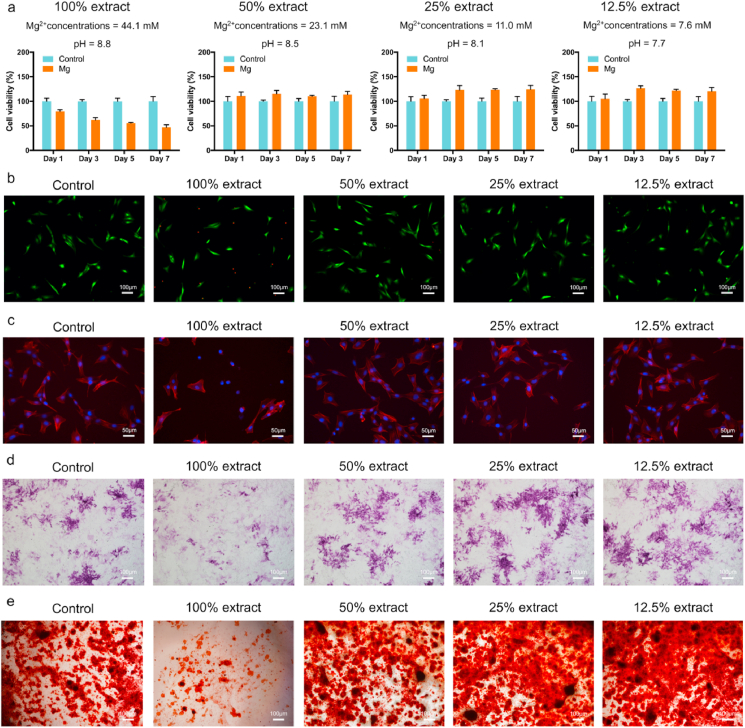
Cytocompatibility and osteogenic differentiation *in vitro*. a) Cell viability, b) live/dead cells, and c) cell morphology of the MC3T3-E1 cells cultured with different extract concentrations. d) ALP staining after 7 d of culture. e) Alizarin red staining of cells cultured with different extract concentrations for 21 d.

The MC3T3-E1 and RAW 264.7 cells were cultured in the presence of different extract concentrations (100, 50, 25, and 12.5%) to evaluate the cytotoxicity of the 3D-printed JDBM implant. A previous study recommended the use of 6–10 dilutions of extracts to the perform *in vitro* cytotoxicity testing [29]. First, we evaluated the proliferation of cells cultured with different extract concentrations for 1, 3, 5, and 7 d (Fig. 3a and Fig. S4a). The 50, 25, and 12.5% sample extracts did not inhibit the viability of the MC3T3-E1 and RAW 264.7 cells. In addition, the 50, 25, and 12.5% sample extracts did not increase the number of dead cells (Fig. 3b and Fig. S4b) and did not change cell morphology (Fig. 3c and Fig. S4c). Therefore, 3D-printed JDBM implants have *in vitro* cytocompatibility.

### 3.4 Osteogenic differentiation

The osteogenic differentiation of pre-osteoblasts cultured in different extract concentrations (100, 50, 25, and 12.5%) was determined by ALP staining and alizarin red staining. As demonstrated by ALP staining (Figs. 3d), 25 and 12.5% of the sample extract enhanced the osteogenic differentiation of MC3T3-E1 cells at the earlier stage. Furthermore, alizarin red staining confirmed that the 25 and 12.5% sample extracts promoted calcified nodule formation after 21 d of culture (Fig. 3e), suggesting that 3D-printed JDBM implants had potential osteogenic properties.

### 3.5 In vitro antibacterial performance

**Fig. 4:**
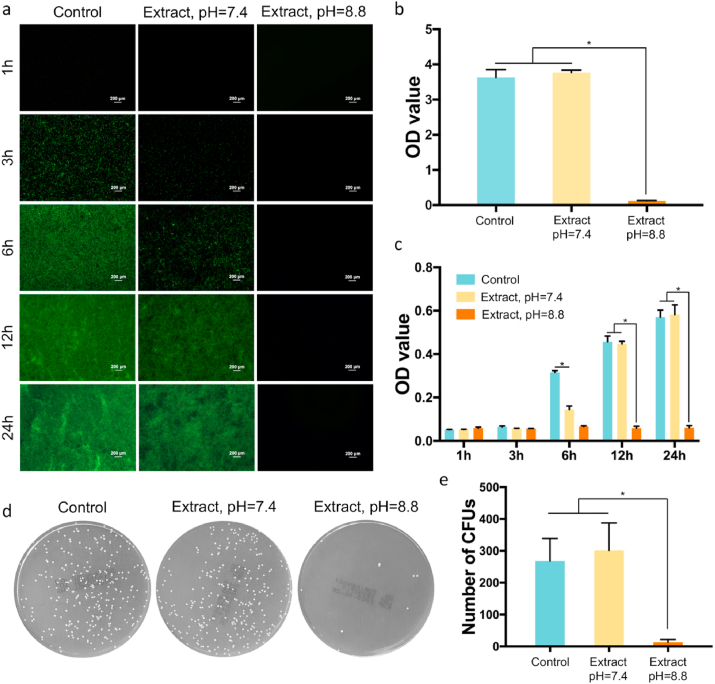
Antibacterial activity of 3D-printed JDBM implants *in vitro*. a) Biofilm formation of MRSA cultured in different media after 1, 3, 6, 12, and 24 h of incubation. b) Absorption of crystal violet by MRSA biofilm after 24 h of incubation. c) Semi-quantitative analysis of bacterial concentration after 1, 3, 6, 12, and 24 h of incubation. d) Representative images of MRSA cultured in different media. e) Statistical analysis of colony number after 24 h of incubation. *p < 0.05.

Based on the LIVE/DEAD staining, the sample extract (pH = 8.8) showed significant anti-biofilm properties (Fig. 4a and Fig. S5a). Decreased bacterial biofilm formation was observed after 24 h of incubation, according to semi-quantitative analysis of crystal violet staining (Fig. 4b and Fig. S5b). The antibacterial ratios of sample extracts against MRSA and *E. coli* were 90.0% and 92.1%, respectively. As shown in Fig. 4a, bacterial colonies were detected in TSB and sample extract (pH = 7.4, Mg^2+^ concentration = 38.3 ± 2.0 mM) after 3 h of incubation, and the number of adherent bacteria increased with the incubation time, according to the semi-quantitative analysis of bacterial concentration. However, the number of adherent bacteria in the sample extract (pH = 7.4) was lower compared to TSB at 3 and 6 h, suggesting that the high-concentration Mg^2+^ could inhibit the bacterial adhesion of MRSA at low bacterial concentrations (Fig. 4b). In addition, high-concentration Mg^2+^ also disrupted the colonies of MRSA. After 6 h of incubation, the OD value of the bacterial suspension in the sample extract (pH = 7.4) was significantly lower compared to TSB (Fig. 4c). The results of semi-quantitative analysis indicated that the bacterial concentration increased with incubation time (Fig. 4c); however, the antibacterial properties of high-concentration Mg^2+^ decreased as the bacterial concentration increased. The bacterial colony formation of MSRA in sample extract (pH = 7.4) was comparable to that in the control group, after 24 h of incubation (Fig. 4d and e). The sample extract (pH = 8.8) inhibited the colony formation of *E. coli*, according to semi-quantitative analysis of bacterial concentration (Fig. S5c) and spread plate analysis (Figs. S5d and S5e).

### 3.6 In vivo antibacterial performance

#### 3.6.1 Radiographic analysis

**Fig. 5:**
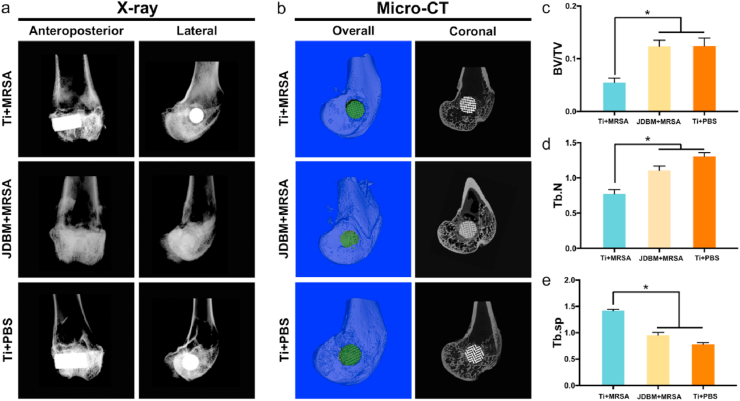
Radiological evaluation of 3D-printed JDBM implants antibacterial activity. a) Anteroposterior and lateral X-ray images of distal femur. b) μCT evaluation of implant-related infections, four weeks after implantation; the parameters included were c) BV/TV, d) trabecular number (Tb.N), and e) Tb.Sp. *p < 0.05.

The anteroposterior and lateral radiographs of the right femur of experimental animals were acquired four weeks after implantation. A progressive implant-related osteomyelitis with severe bone destruction, osteomyelitic sequestration, and periosteal reaction were observed in the Ti+MRSA group; however, no signs of local implant-related infection were detected in the JDBM+MRSA group (Fig. 5a). Bone resorption in the peri-implant tissue was also observed in the 3D reconstruction of micro-CT scanning (Fig. 5b). The quantitative analysis of the 2D images showed that the BV/TV and trabecular number (Tb.N) of the Ti+MRSA group were significantly lower than those in the JDBM +MRSA group (Fig. 5c and d), while the Tb.Sp of the Ti+MRSA group was significantly higher compared to the JDBM+MRSA group (Fig. 5e).

#### 3.6.2 Histological evaluation

**Fig. 6:**
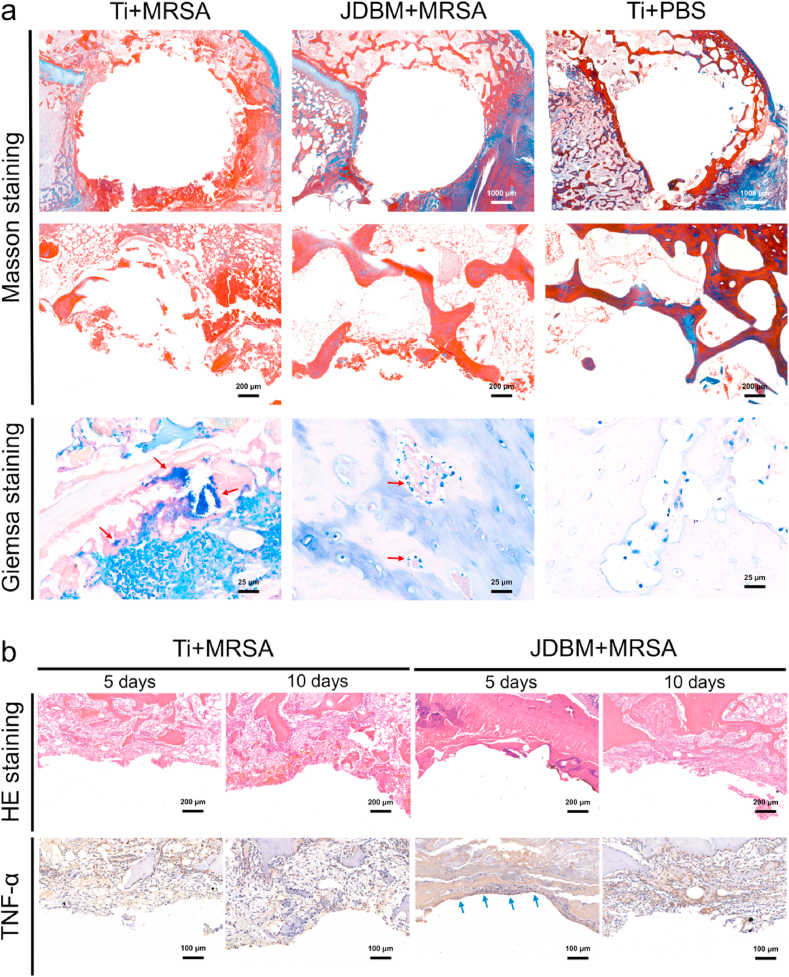
Histological evaluation of implant-related infection. a) Masson and Giemsa staining of peri-implant tissue at four weeks after implantation. b) HE staining and immunohistochemical detection of TNF-α secretion at the bone-implant interface at 5 and 10 days after implantation.

Giemsa staining was used to detect the MRSA colonies in the infected bone, and a large number of bacterial colonies was observed in the peri-implant bone tissue (red arrows in Fig. 6a, Giemsa staining, Ti+MRSA group). In contrast, only a small number of bacteria was observed in the JDBM+MRSA group (red arrows in Fig. 6a, Giemsa staining, JDBM+MRSA group), suggesting that 3D-printed JDBM implants could prevent implant-related infection *in vivo*. Peri-implant bone tissue destruction with severe inflammatory cell infiltration was observed in the Ti+MRSA group (Fig. 6a), confirming MRSA-induced implant-related infection. A low-level inflammatory response was observed in the JDBM+MRSA group, and the structure of peri-implant bone tissue was comparable to that of the negative control at 4 weeks after implantation (Fig. 6a and Fig. S6). However, enhanced inflammatory cell infiltration and increased TNF-α secretion were observed at the bone-implant interface during the early implantation stage (day 5 after surgery) in the JDBM+MRSA group (Fig. 6b), which was inconsistent with peri-implant inflammatory response at 4 weeks after implantation. Since it is difficult to quantify the Mg^2+^ release *in vivo*, the degradation rate and Mg^2+^ release were evaluated in our *in vitro* experiments. As shown in Fig. 2e, the Mg^2+^ concentration increased rapidly during the first 6 days. Based on our results and previous studies [20,24,34,35], we hypothesized that the temporally enhanced peri-implant immunological response was caused by relatively high Mg^2+^ release, which could be beneficial for the inhibition of implant-related infection.

### 3.7 Effects of high-concentration Mg^2+^ on macrophage function

**Fig. 7:**
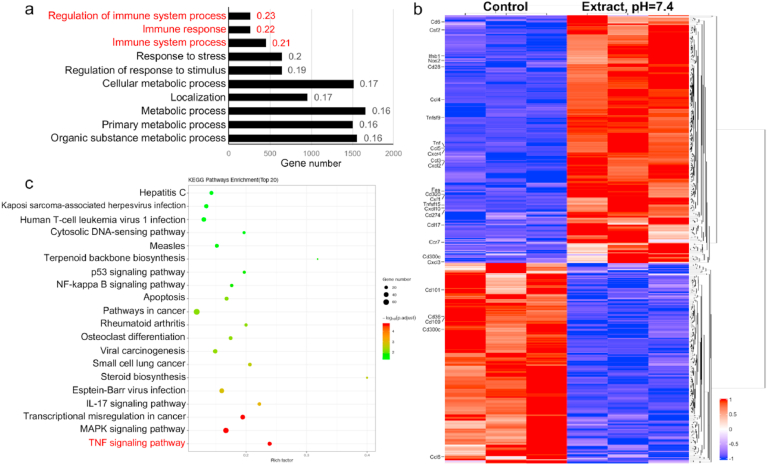
Transcriptomic analysis of macrophages incubated with high Mg^2+^ extracts. a) GO enrichment analysis of biological processes for the differentially expressed genes induced by high Mg^2+^ extracts after 3 days of incubation. b) Differentially expressed genes in the GO terms of the regulation of immune system process, immune response, and immune system process. c) KEGG enrichment analysis of the pathways involved in the immunoregulatory effect induced by high Mg^2+^ treatment.

Next, to determine the effect and underlying mechanisms of high Mg^2+^ (sample extract, pH = 7.4, Mg^2+^ concentration = 43.3 ± 1.8 mM) on macrophage function, we performed a transcriptomic analysis. Compared to the control, 4466 differentially expressed genes were identified (a threshold with absolute log2 fold change >1 and p < 0.05). The GO analysis revealed that these differentially expressed genes were related to biological processes involved in the antibacterial activity of macrophages, such as the regulation of immune system process, immune response, and immune system process (Fig. 7a). The analysis of these three biological processes showed that the expression of M1-related genes (*Tnf*, *iNos*, *Ccl3*, *Ccl4*, *Ccl5*, *Cxcl10*, and *Cxcl2*) was induced (Fig. 7b). Furthermore, KEGG enrichment analysis indicated that the TNF signaling pathway was greatly upregulated by the high-concentration Mg^2+^ treatment (Fig. 7c).

**Fig. 8:**
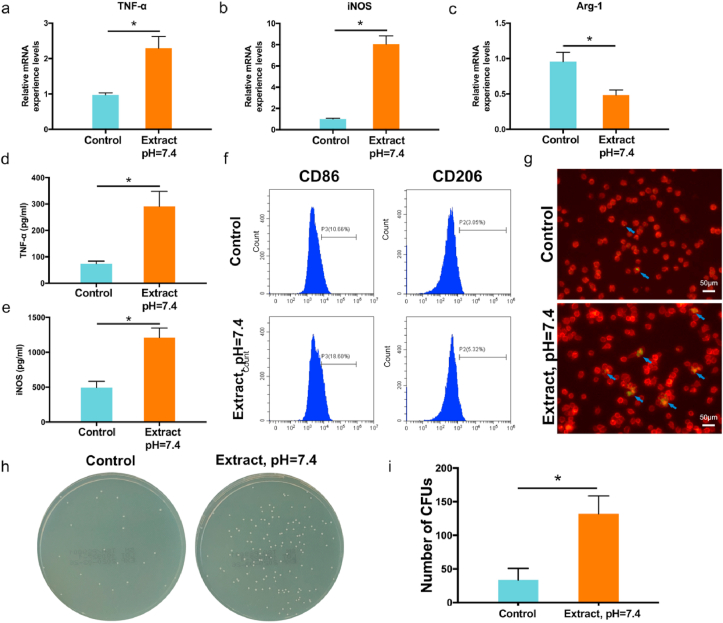
Immunoregulatory antibacterial effect of macrophages in high Mg^2+^ environment caused by degradation of the 3D-printed JDBM implant. Relative mRNA expression levels of a) TNF-α, b) iNOS, and c) Arg-1 genes. Cytokine concentration of d) TNF-α and e) iNOS determined by ELISA. f) Evaluation of macrophage polarization markers CD86 and CD206 by flow cytometry. g) Fluorescence staining (blue arrows) of MRSA phagocytosis. h) Representative images of phagocytosed MRSA detected by spread plate analysis. i) Quantification of phagocytosed MRSA, colony number. *p < 0.05.

The expression of TNF and iNOS genes was further validated at the mRNA level, using RT-PCR. After 72 h of incubation, high-concentration Mg^2+^ stimulated the expression of TNF-α and iNOS genes (Fig. 8a and b). In addition, the expression of the *Arg-1* (M2 phenotype marker) was downregulated (Fig. 8c). At the protein level, high-concentration Mg^2+^ also induced the expression of TNF-α and iNOS in macrophages, as demonstrated by ELISA (Fig. 8d and e). Flow cytometry showed that the percentage of CD86 positive RAW 264.7 cells (M1 phenotype) increased from 10.66% to 18.60% after 72 h of incubation with the sample extract (pH = 7.4, Fig. 8f). Therefore, these results indicated that high Mg^2+^ concentrations in the sample extract promoted macrophage polarization into the microbicidal M1 phenotype.

The enhanced M1 macrophage polarization also improved the phagocytic ability of the RAW 264.7 cells. Fluorescence staining showed that the levels of phagocytosed MRSA increased after the high-concentration Mg^2+^ treatment (Fig. 8g). The phagocytic ability of macrophages was further validated by spread plate analysis (Fig. 8h and i). A higher number of bacterial colonies was observed in the JDBM group, indicating that these macrophages had higher levels of phagocytosis activity compared to the control group.

### 3.8 Biosafety evaluation

The Mg^2+^ deposition in major organs of experimental animals was determined using the ICP-MS analysis (Table S2). There was no significant difference in Mg^2+^ concentration in the heart, liver, spleen, lung, and kidney between the healthy control and JDBM groups. The serum Mg^2+^ concentration in the JDBM group was comparable to that in the control group (Fig. S7). In addition, hepatic and renal function damage was not observed in experimental animals implanted with 3D-printed JDBM implants, as evaluated by blood biochemical tests (Table S3). The organizational microstructures of the heart, liver, spleen, lung, and kidney of experimental animals were assessed by HE staining, and no pathological changes in the morphology of major organs were observed in the JDBM group (Fig. S8).

## 4 Discussion

Here we characterize a porous JDBM implant that was fabricated using SLM from the Mg–Nd–Zn–Zr powder. First, we evaluated the antibacterial properties and biocompatibility of 3D-printed JDBM alloy implants *in vitro* and *in vivo*. Next, we confirmed that 3D-printed JDBM alloy implants could protect against MRSA, decrease biofilm formation, and prevent MRSA-induced implant-related infection in a rabbit model. The high concentration of Mg^2+^ produced by the degradation of 3D-printed JDBM alloy implants was not only responsible for bacterial inhibition, but also had immunoregulatory antibacterial activity by promoting M1 macrophage polarization. Therefore, the 3D-printed JDBM alloy implant showed biocompatibility, as demonstrated by systematic evaluations both *in vitro* and *in vivo*.

For Mg-based orthopedic implants, the implant design is mainly dependent on the application. For example, cast Mg screws with higher mechanical strength (especially for shear and torsional rigidity) are used for internal fixation of fracture. However, the degradation rate of solid Mg screws does not match the rate of bone repair. As we previously reported, the JDBM screw (3.5 mm diameter) maintained their morphology for 1 year of implantation in patients with malleolar fractures [36]. The relatively low degradation rate can promote the osteogenic activity and biocompatibility of the JDBM screw and is beneficial for maintaining mechanical strength *in vivo*. In contrast, relatively high degradation rate may continuously create an alkaline environment in peri-implant tissue, which can help to improve the antibacterial and anti-tumor effect of the Mg implant. We believe that the ideal degradation rate and favorable mechanical properties of the Mg implant are not contradictory. First, alloying is an effective method for improving both the corrosion resistance properties and mechanical properties of the Mg implant [27]. We previously reported the development of Mg-Nd-Zn-Zr alloy (JDBM) with enhanced mechanical and corrosion resistance properties [37]. Coating can prevent corrosion of the Mg alloy without affecting the mechanical properties. In our previous study, the biodegradable Ca–P coating decreased the degradation rate of JDBM from 0.54 to 0.39 mm/y in Hank's solution [38]. For the 3D-pinted Mg implant, we improved corrosion and mechanical properties by optimizing the processing parameters. In addition, the surface area-to-volume ratio can affect the degradation rate of porous Mg implants, which can be optimized by controlling the pore structure, pore size, and porosity. Therefore, both the degradation and mechanical properties are expected to be manipulated to meet clinical requirements.

Recent advances in metal additive manufacturing techniques offer a novel approach to the fabrication of porous Mg implants with complex shapes. However, the additive manufacturing of Mg alloy implants remains challenging and relatively dangerous due to the highly active chemical properties of Mg [19]. During the SLM process of fabricating Mg alloy, serious spattering is much more common compared to steel, titanium, and aluminum alloys. Furthermore, layer cracks are frequently observed in some 3D-printed Mg alloys [39]. However, as a result of process optimization (laser power, scanning speed, layer thickness, and hatch spacing), such layer cracks were not observed in the 3D-printed JDBM implants. To the best of our knowledge, this is the first study reporting the manufacturing and biological evaluation of 3D-printed Mg (Mg–Nd–Zn–Zr) implants. This 3D-printed JDBM implant had a fully interconnected porous structure, with 324.6 ± 25.7 μm pore size, the size suitable for bone and blood vessel ingrowth [40,41]. The ideal pore structure of 3D-printed Mg implant has not been determined yet. Here in this study, we used the diamond unit cell, similar to the one reported in our previous animal studies and clinical evaluation of 3D-printed porous titanium alloy implants [40,42,43]. Other research groups have also shown bone ingrowth into 3D-printed porous titanium alloy implants with diamond unit cell [44,45]. We have not determined the best pore structure for 3D-printed JDBM implants, and future studies are needed to investigate the effect of pore structure on the mechanical characterization and biological activity of 3D-printed JDBM implants.

Here we used additive manufacturing and showed that this approach generated favorable mechanical properties compared to the soluble-template approach. Jia et al. [46] fabricated porous Mg implants using a soluble NaCl template, and the yield strength of these porous Mg implants ranged from 0.86 ± 0.05 to 1.35 ± 0.17 MPa. For the titanium space holder approach, the compressive yield strength of the porous Mg implant ranged from 4.3 to 6.2 MPa [16]. The compressive yield strength of the 3D-printed JDBM implant was 54.80 ± 6.43 MPa, which was significantly higher than that of the soluble-template approach.

The bacterial killing ability of Mg alloy implants has been widely recognized. Previous studies have reported the antibacterial properties of Mg alloys both *in vitro* and *in vivo* [14,47,48]. However, the mechanism of Mg alloy antibacterial action remains controversial. Our results indicated that the antibacterial properties of Mg alloy implants mainly relied on the alkaline microenvironment at high bacterial concentrations, which is consistent with previous studies [[49], [50], [51]]. Given the pH-dominant antibacterial properties of Mg alloys, there is concern that the buffering ability of the body fluids could reduce the antibacterial effect by neutralizing the alkaline microenvironment *in vivo* [52]. In addition, the degradation rate of Mg alloys in different environments would change due to the variations in the chloride ion concentration, chemical composition, and water content [53,54]. Furthermore, the reduced degradation rate of Mg alloys in bone could decrease the antibacterial properties of the Mg alloys *in vivo* [55]. Compared to solid implants, the large surface area-to-volume ratio of 3D-printed porous Mg implants would increase the degradation rate, which would help to maintain the alkaline environment in the peri-implant bone tissue. Therefore, based on our results, the 3D-printed JDBM implant exhibited antibacterial performance both *in vitro* and *in vivo*.

Implant-related infection is a complicated process that involves the implant, the host immune system, and the bacteria [56]. When host tissues become invaded by bacteria, macrophages are recruited to the infected site and play an essential role in the host's immune response. Macrophage depletion will inevitably increase host susceptibility to pathogens and delay bacterial clearance [57]. Activated macrophages are commonly classified into pro-inflammatory M1 phenotype and anti-inflammatory M2 phenotype. M1 macrophages secrete pro-inflammatory cytokines and participate in the immune defense against bacteria. However, bacteria and bacterial biofilms have been shown to negatively regulate the macrophage-mediated immune defense by promoting macrophage polarization towards the anti-inflammatory M2 phenotype. Immune evasion caused by biofilms is a complex process responsible for difficulties associated with the treatment of implant-related infections [21]. Therefore, developing an immunomodulatory biomaterial that enhances the immune defense against bacteria would be beneficial for preventing and treating implant-related infections [24]. The exact mechanism of the immunomodulatory effect of Mg implants is still not well understood. For example, Mg^2+^ presumably exhibits anti-inflammatory effects at relatively low concentrations. Hu et al. [58] reported that the supplementation of 5 mM in culture medium reduced the mRNA expression of TNF-α, IL-6, and IL-1β in macrophages stimulated with LPS and IFN-γ by inhibiting NF-κB activation. In addition, Mg^2+^ could suppress the function of osteoclasts and attenuate particle-induced osteolysis in a mouse model [59]. Hypomagnesemia promotes chronic low-grade inflammation, and the levels of inflammatory cytokines (TNF-α and IL-6) are increased in Mg-deficient rodents [60,61].

However, the immunomodulatory effect of Mg^2+^ could be completely different in mammalian cells cultured in high concentrations of Mg^2+^. Previous studies have reported that a high concentration of Mg^2+^ promotes inflammatory responses in human umbilical vein endothelial cells and macrovascular endothelial cells by inducing the synthesis of nitric oxide (NO) [34,35]. Our study confirmed the dose-dependent immunomodulatory effect of Mg^2+^. The high Mg^2+^ concentration caused by the relatively fast degradation of the 3D-printed JDBM implant showed the upregulation of proinflammatory responses. The sample extract (pH = 7.4) upregulated the TNF signaling pathway and increased the percentage of pro-inflammatory M1 phenotype *in vitro*, resulting in the enhanced phagocytic ability of macrophages (Fig. 8). Consistent with these data, we observed enhanced immunological response and TNF-α secretion at the bone-implant interface at the early implantation stage (day 5) in the rabbit experiments. Furthermore, the concentrations of TNF-α and iNOS were significantly increased in the RAW 264.7 cells cultured with high Mg^2+^. The inducible iNOS produces high levels of NO and plays an important role in host immunity. For example, NO is essential for bacterial clearance in infectious arthritis [62], while mice lacking iNOS are more susceptible to bacterial infections [63]. Moreover, the development of NO-releasing biomaterials has been confirmed as another potential approach to eliminate bacterial infections [64,65]. A previous study also reported that the rapid degradation of Mg alloy induced macrophage infiltration and enhanced the inflammatory response in peri-implant tissues [20]. We believe that the large surface area-to-volume ratio would increase the degradation rate of 3D-printed JDBM implants, which would help to maintain the high Mg^2+^ environment in peri-implant bone tissues and induce the antibacterial activity of macrophages.

Conversely, the over-activated inflammatory response caused by Mg implants could have a negative effect on bone repair. However, the formation of a corrosion layer would reduce the degradation rate of Mg implants with time, resulting in the reduction of inflammatory response caused by high Mg^2+^. As anticipated, only a low-level inflammation was observed in the peri-implant tissues of the animals in the JDBM+MRSA group after four weeks, while the appearance of the peri-implant bone tissue was comparable to that of the negative control. Finally, 3D-printed JDBM implants exhibited biosafety *in vivo*. The deposition of Mg^2+^ ion complexes in major organs and pathological changes in the microstructures of major organs were not observed in the JDBM group. In summary, our results indicate that 3D-printed JDBM is a biodegradable material with a potential for future clinical application as an orthopedic implant. Further studies are required to optimize the degradation and mechanical properties according to clinical requirements. Moreover, cardiovascular, vascular, tracheal, and ureteral stents are some of the important medical targets that can be generated via 3D-printed Mg devices.

## 5 Conclusion

Here we developed a porous JDBM implant with an interconnected porous structure and suitable mechanical properties using additive manufacturing. The 3D-printed JDBM implant showed antibacterial properties against antibiotic-resistant MRSA and prevented MRSA-induced implant-related infection in a rabbit model. The high Mg^2+^ environment generated by the degradation of 3D-printed JDBM implants promoted M1 phenotype polarization of macrophages and enhanced their phagocytic ability. The increased immunoregulatory effect induced by Mg^2+^ release during the early implantation stage is a potential antibacterial mechanism of Mg-based implants. Additionally, the 3D-printed JDBM implant did not result in any local or systemic toxicity in the major organs. Our results indicate that 3D-printed JDBM implants have a potential for future orthopedic applications.

## CRediT authorship contribution statement

**Kai Xie:** Conceptualization, Investigation, Formal analysis, Writing – original draft. **Nanqing Wang:** Investigation, Formal analysis, Writing – original draft. **Yu Guo:** Investigation, Formal analysis. **Shuang Zhao:** Investigation, Formal analysis. **Jia Tan:** Formal analysis. **Lei Wang:** Methodology, Funding acquisition. **Guoyuan Li:** Methodology, Funding acquisition. **Junxiang Wu:** Investigation. **Yangzi Yang:** Investigation. **Wenyu Xu:** Investigation. **Juan Chen:** Methodology. **Wenbo Jiang:** Methodology. **Penghuai Fu:** Conceptualization, Methodology, Supervision, Funding acquisition, Writing – review & editing. **Yongqiang Hao:** Conceptualization, Supervision, Funding acquisition, Writing – review & editing.

## Declaration of competing interest

All authors have read and approved the manuscript and have no conflicts of interest to disclose.
