# Supplementary material for: Additively manufactured biodegradable porous magnesium implants for elimination of implant-related infections: An in vitro and in vivo study
Source: Bioact Mater. 2021 Jul 6;8:140–52. doi: 10.1016/j.bioactmat.2021.06.032 (PMC8424517; doi:10.1016/j.bioactmat.2021.06.032)
Supplement: Multimedia component 1 [file mmc1.docx]

**Supplementary Material**

**1．Materials and Methods**

**1.1 Post-processing treatment of 3D-printed JDBM implant**

Electrochemical polishing was performed to remove the powder on the surface following manufacturing. A perchloric acid alcohol solution, consisting of 10 vol% perchloric acid and 90 vol% C_2_H_5_OH, was used as polishing solution in the present study. The perchloric acid alcohol solution was mixed at 1,000 rpm using a magnetic stirrer. The solution temperature was controlled below -20℃; the polishing voltage range was 15-20 V, while the polishing current range was 1.5-2.5 A. The polishing time range was 5-20 min. The polishing was stopped when the implant mass was reduced by ~10%. After polishing, the implant was placed into ethanol for ultrasonic cleaning. After polishing, the implants were solution-treated at 520 °C for 2 h.

**1.2 Cell viability**

Alpha modified Eagle’s minimum essential medium (MEM, Hyclone) and Dulbecco’s Modified Eagle Medium (DMEM, Hyclone) supplemented with 10% fetal bovine serum (Gibco) and 1% streptomycin-penicillin (Hyclone) were used as the culture medium for MC3T3-E1 and RAW 264.7 cells, respectively. The corresponding sample extracts were prepared as mentioned above. Furthermore, The cells (5000/well for MC3T3-E1 cell and 1×10^4^/well for RAW 264.7 cell) were seeded in 96-well plates with corresponding culture medium and cultured at 37 °C with 5% CO_2_. After 24 h of incubation, the culture medium was replaced with sample extracts. After one, three, and seven days of incubation, the cell viability was determined using a Cell Counting Kit-8 assay (Dojindo Molecular Technology, Japan) according to the manufacturer’s instructions (n=5). The cells cultured with culture medium extracts were considered as the control group.

In addition, the biocompatibility and morphology of the 3D-printed JDBM implants were determined through LIVE/DEAD staining and F-actin staining. The cells (5×10^4^/well for MC3T3-E1 cells and 1×10^5^/well for RAW 264.7 cells) were seeded in 24-well plates with the corresponding culture medium. After 24 h of incubation, the culture medium was replaced with sample extracts. After 24 h in culture, the culture medium was gently removed. For LIVE/DEAD staining, the cells were stained with a Calcein-AM/PI double staining kit (Dojindo Molecular Technology, Japan). The cells were fixed with 4% paraformaldehyde at room temperature for 15 min to determine the morphology. After fixation, F-actin was stained with rhodamine phalloidin (Cytoskeleton, Inc., USA), and the cell nucleus was stained with DAPI (Sigma, USA).

**1.3 Determination of bacterial biofilm formation**

The diluted bacterial suspension (500 μL, 1×10^6^ CFU/mL) was added to a glass bottom dish. The culture dish was placed at 37 °C for 1, 3, 6, 12, and 24 h. The nonadherent bacteria were gently removed by three PBS washes after incubation. The bacterial biofilms on the culture dish were fixed in 2.5% glutaraldehyde overnight at 4 °C and stained with the LIVE/DEAD combination dye (LIVE/DEAD Backlight Bacteria Viability kit, Invitrogen) according to manufacturer’s instructions. Following the LIVE/DEAD staining, the bacterial biofilm on the culture dish was observed using fluorescence microscopy (Leica Microsystems, Heidelberg, Germany).

For crystal violet staining, the bacterial suspensions (500 μL, 1×10^6^ CFU/mL) were cultured in a single well of the 24-well plates. After 24 h incubation, the nonadherent bacteria were removed gently by three PBS washes after incubation. The bacterial biofilm was stained with 0.1% crystal violet at room temperature for 15 min and solubilized with 95% EtOH. The OD value of the solutions was determined with a microplate reader at 570 nm (n=5).

**1.4 In vivo anti-infective experiments**

The current experiment was approved by the institutional ethics committee of Shanghai Ninth People's Hospital, Shanghai Jiao Tong University School of Medicine, and was performed in compliance with the national laws on animal experimentation. Female adult New Zealand white rabbits (3.2 ± 0.4 kg) were used for the *in vivo* experiments. A longitudinal incision was made at the lateral side of the right knee under general anesthesia. A canal of 5 mm diameter was created on the lateral femoral condyle using a drill, and 10 μL of MRSA suspension (1×10^6^ CFU/mL) or sterile PBS was inoculated into the canal. Ti or JDBM implants were implanted into the canal, and the defect on the lateral femoral condyle was sealed with bone wax. The Ti + PBS group was considered as negative control group. The experimental animals were euthanized four weeks after implantation to evaluate the antibacterial performance of the implant.

The anteroposterior and lateral-view plain radiographs of the right femur were determined using a Faxitron MultiFocus X-ray system for small animals (Faxitron, Bioptics, LLC, USA) according to the manufacturer’s instructions. The femoral specimens were stored in 70% ethanol for micro-CT scanning (μCT 80; SCANCO Medical AG, Bassersdorf, Switzerland) after 72 h fixation in 4% paraformaldehyde. The femoral condyle was considered the region of interest. High-resolution 3D reconstruction of the femoral condyle was also performed. The bone volume/tissue volume (BV/TV), mean trabecular thickness (Tb.Th), and mean trabecular separation (Tb.Sp) were determined were quantitatively analyzed.

The femoral specimens were fixed in 4% paraformaldehyde for 72 h and decalcified using ethylenediaminetetraacetic acid. After dehydration, all decalcified femoral specimens were embedded in paraffin. The samples were cut and subjected to hematoxylin-eosin (HE), Masson trichrome, and Giemsa staining, and immunohistochemical staining for TNF-α (anti-TNF-α, diluted 1:200 [Novus Biologicals, USA]).

**2. Tables**

Table S1 Commercially synthesized primers used in current study

| **Target gene** | **Direction** | **Primer sequence (5’ to 3’)** |
| --- | --- | --- |
| ***TNF-α*** | F  R | CTGAACTTCGGGGTGATCGG  GGCTTGTCACTCGAATTTTGAGA |
| ***iNOS*** | F  R | GGAGTGACGGCAAACATGACT  TCGATGCACAACTGGGTGAAC |
| ***Arg-1*** | F  R | CATATCTGCCAAGGACATCG  GGTCTCTTCCATCACTTTGC |
| **GAPDH** | F  R | CTTCATTGACCTCAACTACATGGTCTA  GATGA CAAGCTTCCC ATTCTCAG |

Table S2 Mg^2+^ deposition in major organs of experimental animal at 4 weeks after implantation (mg/kg).

|  | Heart | Liver | Spleen | Lung | Kidney |
| --- | --- | --- | --- | --- | --- |
| Control | 93.6 $\pm3$.7 | 123.3 $\pm7.0$ | 150.0 $\pm$ 11.3 | $96.9\pm3.6$ | 108.0 $\pm4.6$ |
| JDBM | 99.5 $\pm7.4$ | 123.0 $\pm6.6$ | 163.7 $\pm14.6$ | $86.9\pm8.5$ | 107.0 $\pm14.1$ |
| p value | 0.442 | 0.965 | 0.300 | 0.072 | 0.981 |

Table S3 Results of blood tests of experimental animal at 4 weeks after implantation.

|  | **Cr** | **ALT** | **AST** | **ALB** | **A/G** |
| --- | --- | --- | --- | --- | --- |
| **Control** | 69.2 $\pm$9.2 | 39.8 $\pm14$.4 | 21.1 $\pm7.4$ | 49.3 $\pm7.0$ | 2.2 $\pm0.8$ |
| **JDBM** | 76.6 $\pm$9.4 | 37.4 $\pm$ 9.7 | 21.2 $\pm$ 4.5 | 47.4 $\pm2.5$ | 2.5 $\pm0.8$ |
| **p value** | 0.363 | 0.771 | 0.972 | 0.652 | 0.710 |

**3. Figures**

**
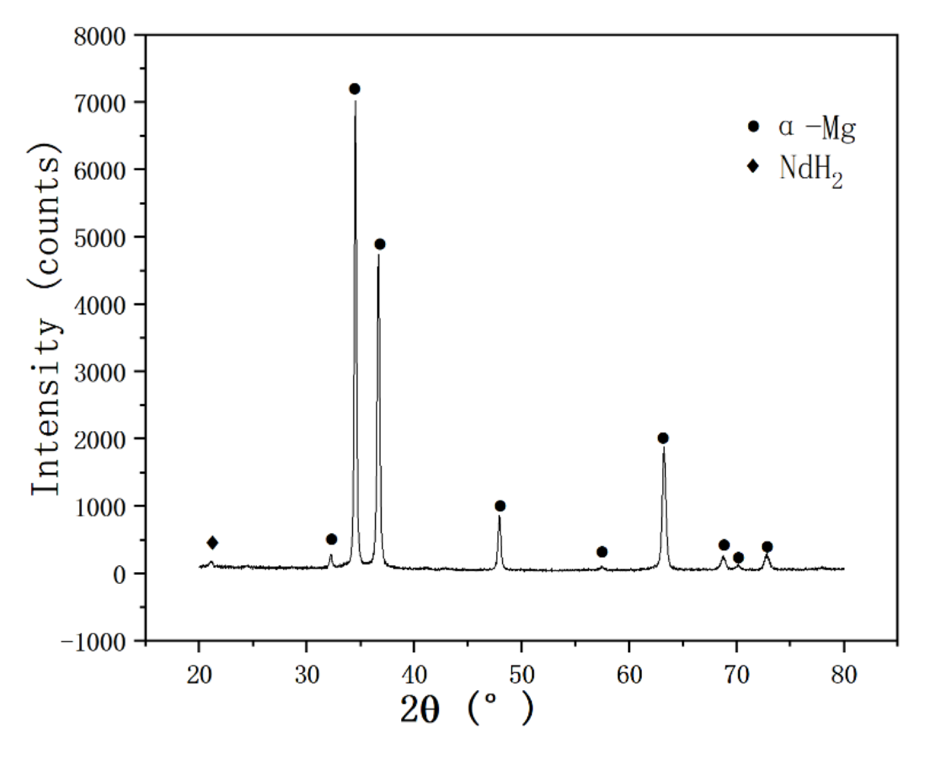
**

Fig. S1. XRD results showing phase constitution of as-fabricated implant.

**
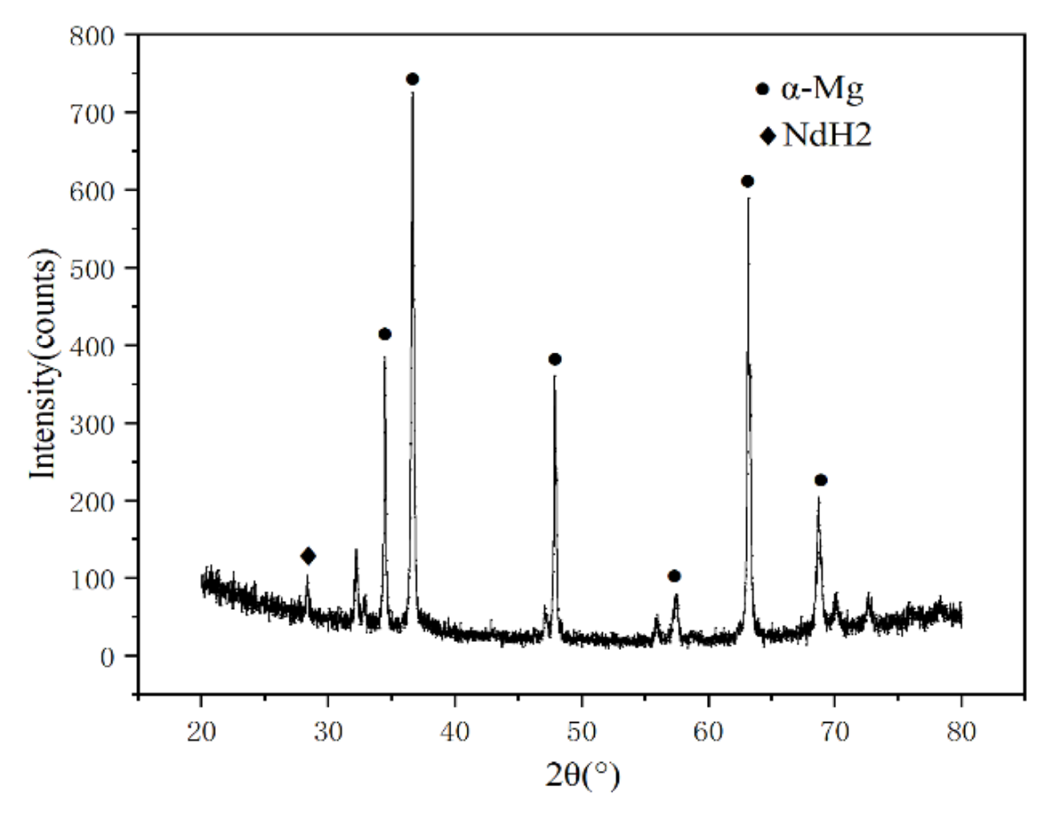
**

Fig. S2. XRD curve of 3D-printed JDBM implant after solution treatment

**
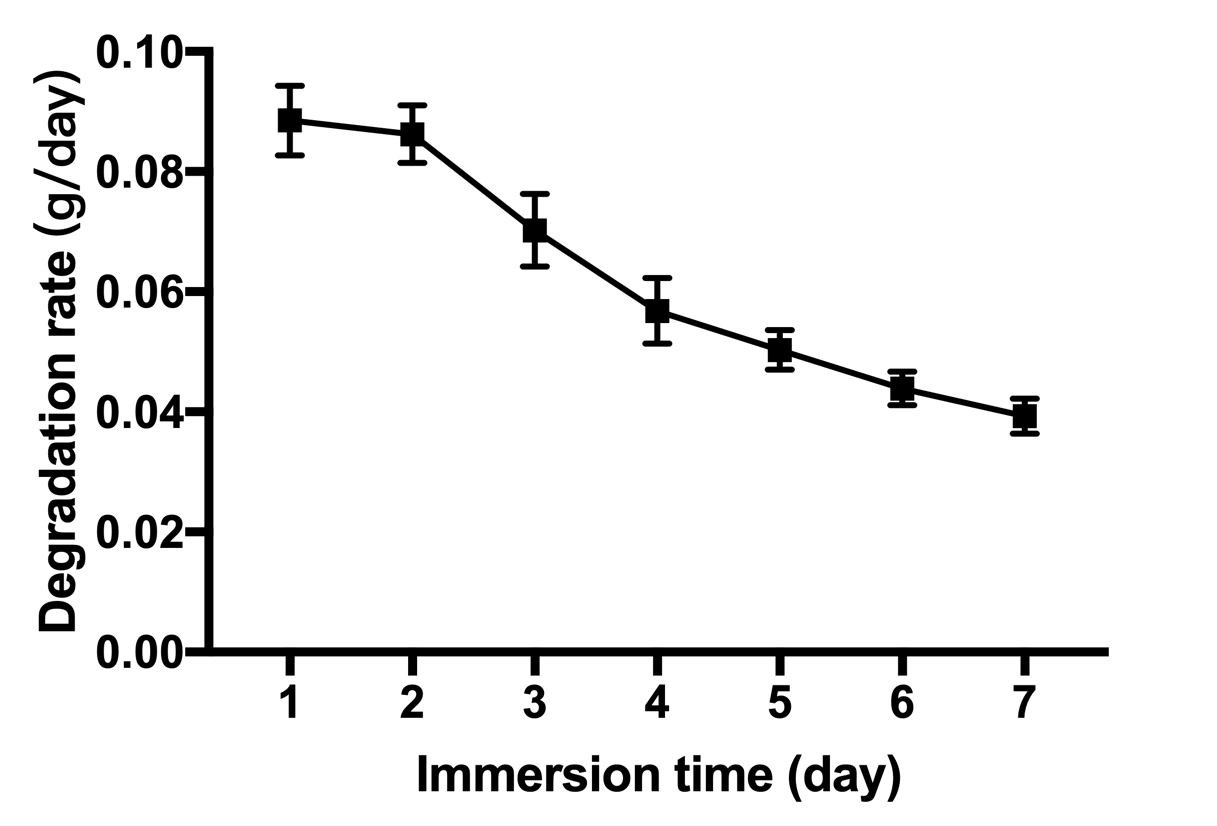
**

Fig. S3. The degradation rate of 3D-printed JDBM implant after immersing for 1–7 days.


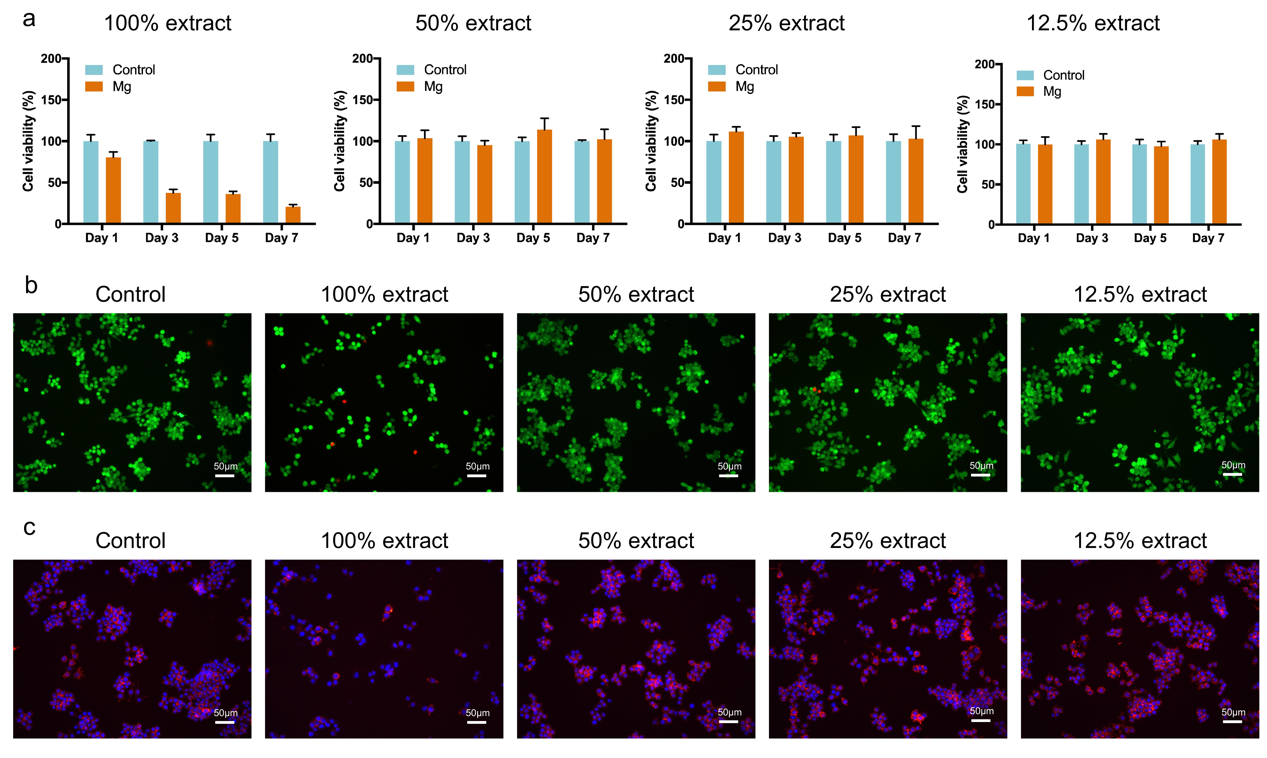
Fig. S4. The evaluation of cytocompatibility with RAW 264.7 in vitro. The cell viability a), live/dead cells b), and cell morphology c) of macrophages cultured in sample extract at different concentrations.


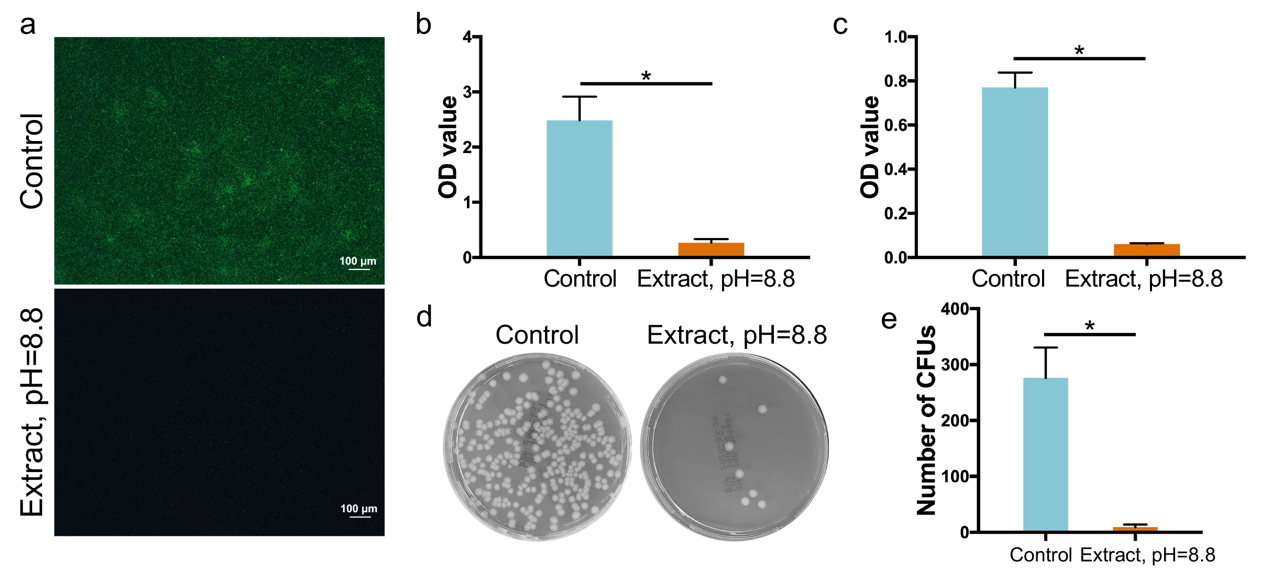


Fig. S5. Antibacterial activity of 3D-printed JDBM implants in vitro. a) Biofilm formation of *E. coli* cultured in different media after 24 h of incubation. b) Absorption of crystal violet after 24 h of incubation, c) Semi-quantitative analysis of bacterial concentration after 24 h of incubation. d) Representative images of *E. coli* cultured in different media. e) Statistical analysis of colony number after 24 h of incubation. *p < 0.05.


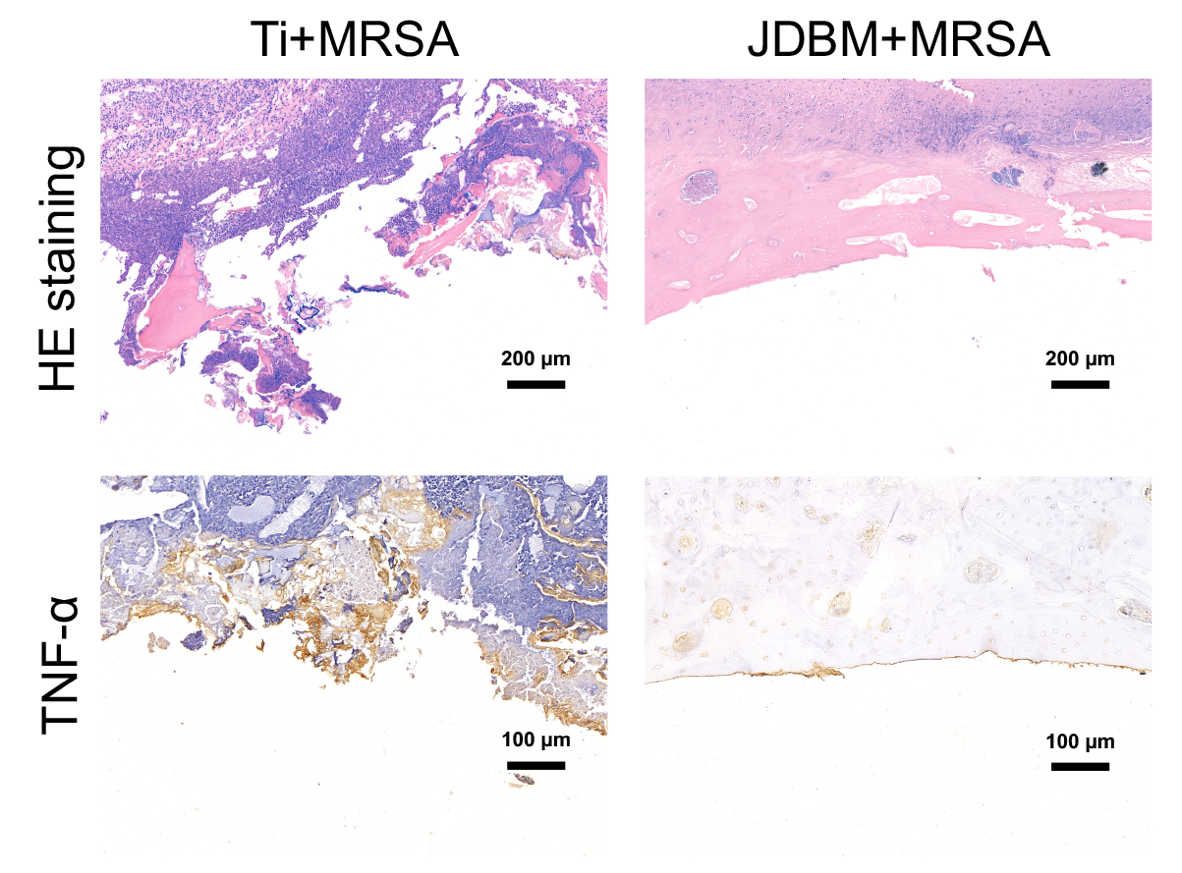


Fig. S6. HE staining and immunohistochemical detection of TNF-α secretion at bone-implant interface at 4 weeks after implantation.


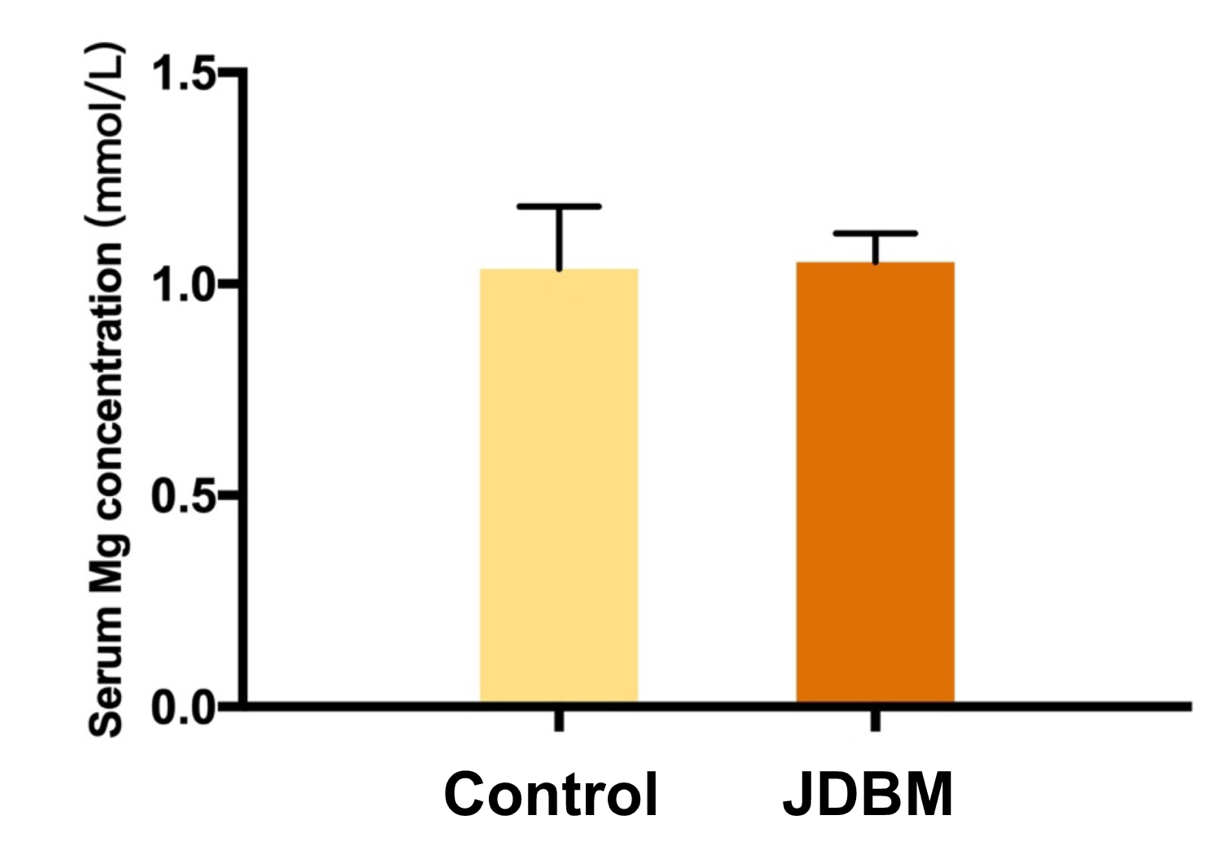


Fig. S7. The serum Mg concentration of experimental animals in Mg groups and Control group at 4 weeks postoperatively.


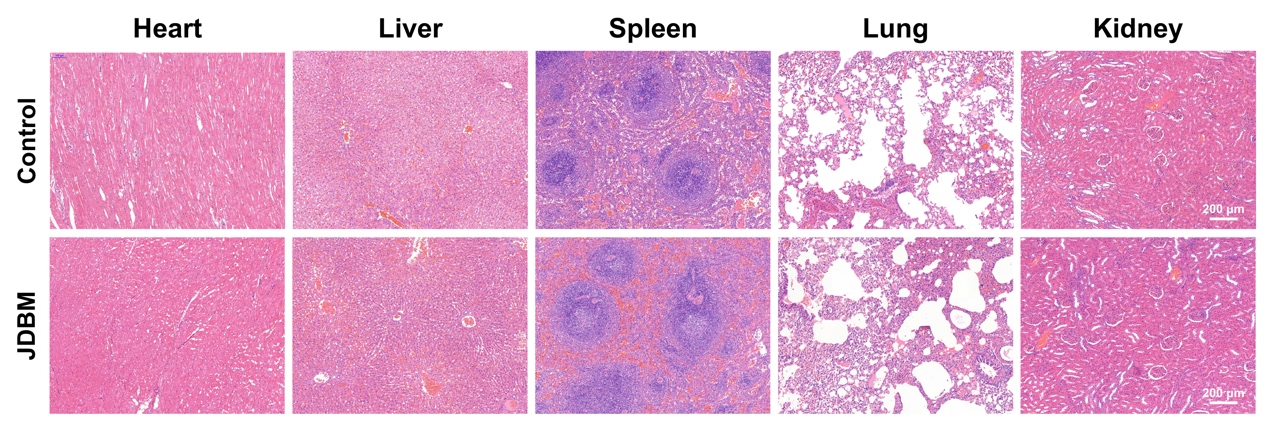


Fig. S8. Evaluation of organizational microstructures of the heart, liver, spleen, lung, and kidney with HE staining.
